# Nurses’ perceptions of and satisfaction with the use of automated dispensing cabinets at the Heart and Cancer Centers in Qatar: a cross-sectional study

**DOI:** 10.1186/s12912-015-0121-7

**Published:** 2016-01-14

**Authors:** Manal Zaidan, Fatma Rustom, Nancy Kassem, Sumaya Al Yafei, Linda Peters, Mohamed Izham M. Ibrahim

**Affiliations:** Pharmacy Department of NCCCR, and Pharmacy Department of Heart Hospital, Hamad Medical Corporation, Doha, Qatar; Pharmacy Department, Heart Hospital, Hamad Medical Corporation, Doha, Qatar; Pharmacy Department NCCCR, Hamad Medical Corporation, Doha, Qatar; Nursing Department, Heart Hospital, Hamad Medical Corporation, Doha, Qatar; Social & Administrative Pharmacy, College of Pharmacy, Qatar University, PO Box 2713, Doha, Qatar

**Keywords:** Automated dispensing cabinet, Hospital Pharmacy, Nurses, Perception, Satisfaction, Qatar

## Abstract

**Background:**

Automated dispensing cabinets (ADCs) were introduced in 2010 and 2012 at the Heart Hospital (HH) and National Center for Cancer Care and Research (NCCCR), both run by Hamad Medical Corporation in Qatar. These medication distribution systems provide computer-controlled storage, dispensing, and tracking of drugs at the point of care in patient care units. The purpose of this study was to assess nurses’ perceptions of and satisfaction with the use of ADCs at HH and NCCCR.

**Methods:**

A cross-sectional study was conducted in the two institutions in May and November 2012 using a piloted, validated, online, and anonymous questionnaire. The questionnaire consisted of four parts: nurses’ sociodemographic and practice characteristics, 21 questions about their perceptions, one question about their overall satisfaction, and one about the system’s ease of use. The self-administered survey was distributed to 503 nurses working at HH and NCCCR over three weeks using Survey Monkey®.

**Results:**

The survey response rate was 80 % (*n* = 403). No significant difference was found in perception scores between the two institutions (*p* = 0.06). Ninety-four percent (*n* = 378) of nurses agreed that the medication delivery system allowed them to do their job more safely, and 90 % (*n* = 363) nurses agreed that they now spent less time waiting for medication from the pharmacy than they did before the ADC system was introduced. Eighty seven percent (*n* = 349) nurses agreed that they were able to administer medication more efficiently with the ADC system. The overall satisfaction rate (either “very satisfied” or “satisfied”) for the two hospitals was 91 %.

**Conclusions:**

The nurses’ perceptions of and levels of satisfaction with the ADC system were very good over the 6 months after complete implementation and integration at HH and NCCCR. ADCs appear to increase efficiency in the medication process and should therefore improve the quality of care.

**Electronic supplementary material:**

The online version of this article (doi:10.1186/s12912-015-0121-7) contains supplementary material, which is available to authorized users.

## Background

According to Fung et al. (2009), improving patient safety is a key focus in hospital settings, and pharmacists have explored a variety of strategies and technologies in support of this aim [1]. Pharmacy practitioners have recommended automated dispensing machines as one possible mechanism to improve patient safety and efficiency [1]. These are decentralized medication distribution systems that provide computer-controlled storage, dispensing, and tracking of medicines. Pharmacists are responsible for delivering medication to patients in a proper, precise, and timely way. In acute care settings, distribution systems have been established that enable pharmacists to evaluate patient-specific medication orders. Pharmacists can also monitor the preparation, packaging, selection, and delivery of these orders to patient care units. Pharmacists’ responsibilities to expand the distribution system and improve its efficiency have been helped by technology. For example, automated dispensing cabinets (ADCs) and other similar devices are used increasingly often in healthcare organizations. Interest in the availability and use of these devices has risen because of the emphasis on direct patient care, changes in healthcare systems, and pressure to reduce healthcare costs [2].

Hamad Medical Corporation (HMC) is the principal public healthcare provider for the State of Qatar. The corporation manages eight hospitals along with further specialist clinical, educational, and research facilities. The National Center for Cancer Care and Research (NCCCR), a 62-bed facility, was opened in 2004 and has been accredited by the Joint Commission International (JCI) since 2006. The Heart Hospital (HH) was opened in May 2011, with 120 beds and is currently pursuing JCI accreditation.

Unit-based ADCs were implemented in these two tertiary care specialty teaching hospitals in 2011. Before this, the hospitals within HMC relied heavily on manual medication distribution systems, including traditional floor stock and medication carts, which held a 24-h supply of patient-specific medications in individual patient cassettes. A floor stock system is flexible, but the pharmacy has little control over inventory. Although the 24-h unit-dose cart offers tighter inventory control, it is often regarded as an inefficient drug distribution system [3]. As well as being labor intensive, other major concerns with these carts include delays in delivery of the first dose, loss of doses, and “borrowing” of patients’ medication [4].

Nurses are the end users of the ADC system and their opinions and feedback are very important in monitoring the implementation and integration of systems. The impact of the ADC system on patient safety and on working conditions for nursing staff is not well known, even though these systems have been used for many years, especially in North America [5]. This study assessed nurses’ perceptions of and satisfaction with the use of ADC at HH and NCCCR. To our knowledge, it is the first study of its type in the Middle East. A study was conducted in Saudi Arabia, but did not measure users’ satisfaction with the ADC [6].

## Methods

### Study design and location

A cross-sectional study was designed using an anonymous survey for nurses. The survey was conducted using a piloted and validated questionnaire over a 3-week period (Additional file 1). It was administered in May 2012 at the HH and in November 2012 at the NCCCR. The survey was developed by the pharmacists working in the pharmacy department.

### Ethical considerations

This study was approved by the Research Strategy and Assurance Committee at HMC. All nurses were sent an email explaining the purpose of the study, and that they were not obliged to participate. No formal consent form was used, but a returned questionnaire was considered to be implied consent to participate.

### Population and sampling

Eligible study participants were all nurses from HH and NCCCR. There were 503 nurses in total, 350 from HH and 153 from NCCCR. It was therefore feasible to include all the nurses in the two hospitals.

### Instruments

No nursing perception and satisfaction surveys were found at the time of designing the study. Four unpublished but relevant surveys were provided by the vendor of the ADCs, and statements from these were used with modifications. The tool was evaluated by the heads of the pharmacy and nursing departments to establish face validity. They assessed whether the questions effectively captured the focus of the study. They also checked for errors such as confusing, leading, or multiple questions. The pilot survey was sent to 20 nurses chosen at random from among the different units in the Heart Hospital. These nurses were not included in the final survey. Their comments were taken into account to see if any amendments to the survey were necessary.

The survey consisted of four parts: 1) the nurses’ sociodemographic and practice characteristics; 2) 21 questions on their perceptions on safety, training, efficiency, timeliness, availability and accessibility aspects, with a 5-point Likert scale (Strongly Agree = 1 to Strongly Disagree = 5); 3) an overall satisfaction question with a 4-point Likert scale; and 4) questions about the system’s ease of use, with a 4-point Likert scale. The reliability was assessed using Cronbach’s Alpha, which was 0.76 based on the 21 perception statements. The average perception score for the 21 items was established; a lower score indicates a higher rate of agreement.

### Data collection

A link to the survey on Survey Monkey® was distributed to all nurses through their official work e-mails with a 2-week deadline for responses. As a follow-up, and to increase response rate, the deadline was later extended by a week.

### Data analysis

Data from the Survey Monkey® program were exported to SPSS version 20 directly from the online survey. The data were analyzed using descriptive and inferential statistics, including frequency and percentage, Chi-Square test for categorical variables, and independent t test and one-way analysis of variance for continuous data (followed by post hoc test). A normality test was carried out on the perception score. The significance level was set at an alpha level of 0.05.

For the open-ended questions, content analysis was used. Words and phrases in the open-ended responses were analyzed by three team members, and then compared. Words about satisfaction and dissatisfaction were identified and coded. The codes were input into SPSS, and then analyzed.

## Results

### Sociodemographic and practice characteristics

The total response rate was 80 % (*n* = 403/503); 93 % in NCCCR and 75 % in HH. The majority of the nurses who responded were staff nurses (*n* = 345, 86 %), working in medical wards (*n* = 161, 40 %), with 5–10 years of nursing experience (*n* = 144, 36 %) and with either a diploma in nursing (*n* = 201, 50 %) or a Bachelor of Science degree in nursing (*n* = 195, 48 %). Around 96 % (*n* = 386) of the nurses had not worked with an ADC system before joining the institutions. When asked how long they had worked with the ADC system within the institutions, 191 nurses (47 %) said more than 6 months and 181 (45 %) said 3–6 months. Details of their current positions, areas of work, patients per nurse, qualifications, and years of experience are given in Table 1.

**Table 1:** Nurses’ sociodemographic and practice characteristics

Characteristics	Number (%) (*N* = 403)	NCCCR (*N* = 142)	HH (*N* = 261)
Title			
Staff nurse	345 (86)	124 (88)	221 (85)
Charge nurse	42 (10)	13 (9)	29 (11)
Head nurse	11 (3)	2 (1)	9 (3)
Others	5 (1)	3 (2)	2 (1)
Nursing unit			
Medical ward	161 (40)	142 (100)	106 (41)
Intensive care unit	140 (35)	0	118 (46)
Emergency	84 (21)	0	19 (7)
Others	17 (4)	0	17 (6)
Nurse:patient			
1:4	52 (13)	21 (15)	31 (12)
1:3	189 (47)	84 (59)	105 (40)
1:2	93 (23)	37 (26)	56 (22)
1:1	69 (17)	0	69 (26)
Degree			
Diploma in nursing	201 (50)	70 (49)	131 (50)
BSc Nursing	195 (48)	68 (48)	127 (49)
MSc Nursing	5 (1)	4 (3)	1 (0)
Others	2 (1)	0	2 (1)
Years of experience			
<5	87 (21)	31 (22)	56 (21)
5–10	144 (36)	46 (32)	98 (38)
11–15	109 (27)	35 (25)	74 (28)
>15	63 (16)	30 (21)	33 (13)
Have you worked with ADC system before joining HH/ NCCCR?			
Yes	16 (4)		
No	386 (96)		
When have you started using ADC system at the NCCCR/HH?			
>3 months	30 (7)		
3–6 months	181 (45)		
<6 months	191 (48)		
Never used the system	1 (0)		

### Nurses’ perceptions

The total score of the 21 perception statements ranged from 21 points up to 63. The levels of agreement for each statement were numerically coded as Agree = 3, Neutral = 2, and Disagree = 1. The results for each perception statement were consolidated from five to three levels of agreement (agree, neutral, disagree) and the findings are shown in Table 2.

**Table 2:** Perceptions of nurses (*n* = 403) about the ADC system

Statement	Agree N (%)	Neutral N (%)	Disagree N (%)
The medication delivery system allows me to do my job more safely	378 (94)	20 (5)	5 (1)
There are rarely discrepancies when doing narcotic count	304 (75)	65 (17)	34 (8)
I have access to all of the medications I need	292 (71)	42 (10)	69 (19)
I am able to get all of my medications in one place	275 (68)	53 (13)	75 (19)
Medications are more readily available	338 (84)	46 (11)	19 (5)
I rarely have to wait in line to get my patient medications	254 (63)	80 (20)	69 (17)
Refrigerated medications are easily accessible	305 (76)	53 (13)	45 (11)
It is easy to obtain medications during an emergency	259 (64)	67 (17)	77 (19)
The physical layout of the system is user friendly	367 (91)	32 (8)	4 (1)
All drawers’ types assures safety access and removal of medications	346 (86)	39 (10)	18 (4)
I can use the system confidently after minimal training	373 (93)	20 (5)	10 (2)
The training materials provided were informative and adequate	379 (94)	22 (5)	2 (1)
Did you view the ADC video prior to Go-Live?	271 (67)	46 (11)	86 (22)
I was adequately trained by the ADC representative or a super-user nurse prior to Go-Live	358 (89)	30 (7)	15 (4)
The system would work better if more medications were in the ADC system	352 (87)	44 (11)	7 (2)
I am able to administer medications more efficiently (on time, right dose, etc.) with the ADC system	349 (87)	38 (9)	16 (4)
I am able to select the best available strength of ordered medications	357 (89)	27 (7)	19 (4)
I now spend less time waiting for medications that come from pharmacy than before ADC system installed	363 (90)	27 (7)	13 (3)
The amount of time between a written order is sent to pharmacy and when it is available from the ADC system is acceptable	272 (67)	83 (21)	48 (12)
The number of phone calls to the pharmacy requesting missing medications is acceptable	228 (57)	104 (26)	71 (17)
Pharmacy personnel have been responsive in answering questions and/or resolving issues	344 (85)	47 (12)	12 (3)

In general, the levels of agreement with the statements were high. Almost all the nurses (94 %, *n* = 378) agreed that the medication delivery system allowed them to do their job more safely. Over two thirds, 68 % (*n* = 272), agreed that the time between an order being written for a patient and sent to the pharmacy, and its availability in the system was acceptable. Most nurses (87 %, *n* = 349) from both hospitals agreed that they could administer the right dose of medication at the right time, and 91 % (*n* = 367) agreed that the physical layout of the ADC system was user friendly. Three-quarters (*n* = 305) of the nursing staff agreed that the refrigerated medication was easily accessible. Similar numbers (*n* = 304) agreed that there were rarely discrepancies in the controlled medication count i.e., narcotics; this was true for both hospitals. Almost all (90 %, *n* = 363) of the nurses agreed that they now spent less time waiting for non-ADC medication to arrive from the pharmacy. Most nurses felt the training had been adequate, with 93 % (*n* = 373) reporting that they could use the ADC system confidently after minimal training and 94 % (*n* = 379) agreeing that the training materials provided were informative and adequate. The perception results were converted into a score and then compared between demographic and practice characteristics and experiences with the ADC (Table 3). Three factors showed significant differences in perception score: nurse to patient ratio in their unit, nursing degree and duration of using ADC. Nurses with low nurse to patient ratio, diploma degree and experience in using ADC more than 6 months have more positive perception. An independent t test was used to compare the difference between nurses in HH (2.01 ± 0.43)) and NCCCR (2.09 ± 0.46). No significant difference was found.

**Table 3:** Differences in average perception score of nurses (*n* = 403) based on their demographic and practice characteristics and experiences with the ADC

Characteristics	Average perception score	*P* value	Significant post hoc test***
Title			
Staff nurse	2.05 (0.45)	0.45**	
Charge nurse	2.07 (0.41)		
Head nurse	1.87 (0.47)		
Others	1.87 (0.29)		
Nursing unit			
Medical ward	2.00 (0.44)	0.52**	
Intensive care unit	2.01 (0.40)		
Emergency	2.14 (0.50)		
Others	2.17 (0.36)		
Nurse:patient			
1:4	2.23 (0.52)	0.00**	1:4 vs 1:3
			1:4 vs 1:1
1:3	2.02 (0.43)		
1:2	2.08 (0.41)		
1:1	1.91 (0.41)		
Degree			
Diploma in nursing	1.97 (0.42)	0.00**	Diploma vs BSc
BSc Nursing	2.10 (0.46)		Diploma vs others
MSc Nursing	2.28 (0.38)		
Others	2.83 (0.37)		
Years of experience			
<5	2.12 (0.47)	0.19**	
5–10	2.05 (0.45)		
11–15	1.99 (0.44)		
>15	1.99 (0.40)		
Have you worked with ADC system before joining HH/NCCCR?			
Yes	2.03 (0.37)	0.96*	
No	2.04 (0.44)		
When have you started using ADC system at the NCCCR/HH?			
<3 months	2.28 (0.52)	0.01**	<3 months vs >6 months
3–6 months	2.05 (0.41)		
>6 months	1.99 (0.45)		
Never used the system	-		

(*) Student t-test or (**) One-way analysis of variance was used at an alpha level of 0.05; (***) tukey’s test was used

### Ease of use

As a result, 95 % (*n* = 381) found the process from logging in to withdrawing medication either “very easy” (47 %, *n* = 189) or “easy” (48 %, *n* = 192). They also agreed that they had access to all the necessary medication (71 %, *n* = 292). Sixty-eight percent (*n* = 275) agreed that they are able to get all of the medications needed in one place. Sixty-four (*n* = 259) of the nurses agreed that it is easy to obtain medications during an emergency.

### Nurses’ satisfaction

The nurses were also asked about their overall satisfaction with the ADC. Almost all were either satisfied (60 %) or very satisfied (31 %), with only 7 % (*n* = 23) “somewhat satisfied” and one not satisfied.

### Qualitative analysis

The nurses made 806 written comments about their favorite and least favorite aspects of the system, and 403 suggestions for improvement. The most popular aspects were that the ADC system prevented or reduced medication errors (*n* = 48), and the system was accessible and easy (*n* = 132). The least popular aspects were that not all medicines in HMC’s formulary were available in the ADC system (*n* = 50), nurses had to queue to obtain medicines (*n* = 49), there was a restricted override list (*n* = 33), and there was an open matrix drawer system (*n* = 12).

The most frequent suggestions made were to make all HMC formulary medication available in the ADC system (*n* = 60), for the pharmacy to enter prescriptions quickly (*n* = 55), to install more machines in certain units (*n* = 37), and to replace the open matrix drawers with locked-lidded drawers (*n* = 16).

## Discussion

This study was conducted in two of the eight hospitals run by HMC, HH, and NCCCR. It explored nurses’ perceptions of and satisfaction with the use of the ADC system in the two hospitals. The safety characteristics of ADCs have improved progressively over the years, but concerns about their use remain. Such worries include the potential to bypass safety features, managing overrides, queuing, making selection errors, storing high-alert medication, and using unsafe practices for medication removal and transportation to the bedside [7, 8]. It is important to improve working conditions for nurses, as this will improve their satisfaction with their work. The results of this study revealed that, overall, nursing staff were satisfied with the use of the technology and believed it facilitated their work and could contribute to safer healthcare and possible reduction in medication errors and “near misses”.

A cross-sectional study conducted in Canada found similarly positive perceptions. Nurses there considered ADCs made their work easier, and helped to provide safe patient care and reduce medication incidents or errors [5]. The majority of nurses agreed that they could do their job more safely using the ADC system, and that it made their job easier. ADCs can decrease the risk of medication errors, but only when cabinet use is carefully planned, and specific safeguards are consistently available and used. Profiled systems are one of the most important safety enhancements to be made to ADCs during the last decade. This safety feature provides a direct interface between the pharmacy information system and ADCs, so pharmacists can profile, screen, and approve medicines before they are removed from the cabinet for administration [7, 8]. Medicines with similar names or packaging, controlled substances, and high-risk medication can all be separated. High-risk medication can be linked to clinical warnings, and safety updates can be implemented easily across departments using the ADC system [8]. Automated dispensing machines eliminate the dispensing of unused “as-needed” doses, thereby lessening the potential for administration errors [1].

In this study, 48 nurses claimed that the ADCs prevented or reduced medication errors, but the impact was not investigated further, and more studies are needed to verify the claim. Although 86 % of the nurses agreed that all drawer types assured safe access and removal of medications, 12 nurses commented that the open matrix drawers were unsafe, and 16 suggested replacing the open matrix drawers with locked-lidded drawers. These can provide a higher level of security by allowing access to only one pre-selected medication at a time. High-capacity, low-security, matrix drawers, which hold large quantities and allow open access to all medicines in the drawer, should be used only for the lowest-risk medicines that otherwise cannot be stored in sufficient quantities [7]. In the 2007 survey by the Institute for Safe Medication Practices (ISMP), just 50 % of respondents said that ADCs were configured with individual compartments for each drug rather than matrix drawers with access to multiple drugs [7]. This suggests that additional controls and process improvements are needed to reduce risk [8].

The questionnaire contained five statements about training on ADC systems. Nurses agreed that they could use the system confidently after minimal training. The training materials provided were informative and adequate, and the nurses were adequately trained by the ADC representative or another nurse prior to the system going live. The nursing staff also agreed that the pharmacy personnel had been responsive in answering questions and resolving any issues. The ADC system managers at both hospitals had ensured that all nurses received standardized education materials and training, and nurses had only been given access to the system when they had passed a competency assessment. The majority of nurses therefore found the ADC system easy or very easy to use.

The majority of nurses also reported that the physical layout of the system was user friendly. The nurses were able to administer medication more efficiently, on time, and in the right dose using the ADC system. Profiled ADCs ensure that nurses can only administer medication that has been reviewed by a pharmacist. The ADC system also interfaces with the pharmacy management system, electronic medical records, and admission/discharge/transfer and materials management, all of which serves to support the medication process [2].

The majority of the nursing staff in both hospitals reported that there were rarely discrepancies when doing narcotics counts. According to the ISMP, ADCs are designed to contain high-risk medication [7]. Automated dispensing machines provide secure medication storage on patient care units, supported by electronic tracking of the use of narcotics and other controlled drugs. Reports can be generated to help identify and prevent potential problems. Automated dispensing machines save nursing time by eliminating the need for manual end-of-shift narcotic counts in these units [1].

A significant number of nurses agreed that they spent less time waiting for medication from the pharmacy than before the ADC system was installed. When nurses were asked to say what they liked about the system, 58 wrote that they valued being able to administer medication without delay and without waiting for them to be delivered from the pharmacy.

The time elapsing from when an order was written and sent to the pharmacy, and when it was available from the ADC system, was acceptable to nurses. There were 37 written comments that highlighted that the time required to fulfil a prescription was slow, especially for urgent or emergency medication requests. According to HMC’s medication policy, urgent medication should be administered within 30 min of being prescribed by a physician [9]. Pharmacists may therefore need to educate nurses to help them identify when medication is urgent. They should also audit and track urgent and emergency medication orders to review and improve the process.

Although 49 nurses commented that they disliked having to queue for patient medication, close to two-thirds of the nurses responded that they rarely had to do so. Queuing is a major difficulty frequently associated with ADC use. In the ISMP ADC survey (2008a), almost one third of frontline nurses reported always or frequently queuing to access the ADC [7]. Queuing is often a symptom of larger issues that lead to workflow barriers. ADC-generated reports can be used to determine if certain access points are being over-used. Data showing much more activity on one device than another can help support the need to provide additional access points.

The majority of the nurses agreed that the system would work better if the ADC system contained more drugs, and 60 nurses suggested adding all HMC’s formulary medication to the ADC system. About 95 % of formulary medications are available in the ADCs. Both hospitals’ Pharmacy and Therapeutics Committees have established criteria for including medication in the inventory. These include requirements that hazardous drugs, or medications that require extensive dilutions or calculations, should not be part of the ADC standard inventory. ADC system managers should continue to analyze ADC activity reports regularly to determine what drugs are not used often and could be removed from the ADC.

Most nurses in the study agreed that they had access to the medications they need, they were able to get all of their patients’ medication in one place, and that refrigerated medication is easily accessible. Accessibility was valued; 69 nurses commented that they liked the accessibility of the system most of all.

Around two-thirds of the nurses agreed that it was easy to obtain medication during an emergency. Automated dispensing machines enhance first-dose availability and facilitate the timely administration of medication by increasing accessibility on patient care units. This is particularly important in emergency departments and intensive care units [1]. At HH and NCCCR, override medication lists were developed and approved by the relevant Pharmacy and Therapeutics Committee. All medication distribution systems have medication withdrawal functions that allow nurses and other caregivers limited access to certain medications before order review and approval by a pharmacist, especially in cases of patient emergencies. This function is typically referred to as an “override”. Override data evaluation can help hospitals to improve the outcomes of automated dispensing device use by decreasing medication errors and potential adverse drug events. It should therefore be considered part of the routine management process for automated dispensing devices [2]. Nurse training should highlight the risks associated with the override facility.

Finally, the study found that overall, 91 % of nurses across the two hospitals were either very satisfied or satisfied with the use of the ADC system, which is very encouraging.

### Strengths and limitations

As the first peer-reviewed study on this subject in the State of Qatar, and probably in the Middle East, this research has wide-ranging implications. The high response rate (80 %) was helpful to understand how nurses, as the end users, feel about the ADC system.

The study also had several limitations. It was conducted in only two hospitals, both of which are specialist hospitals (for heart and cancer patients). The sample size was not calculated. The findings can therefore not be generalized to other populations or settings in Qatar, or more widely. The study was a post-implementation survey and there was no information about the situation before implementation. No pre-post assessment was therefore possible.

### Implications for practice

The change in the pharmacy distribution model with the use of the ADC system has had broad implications for the working practices of pharmacists, pharmacy technicians, and nurses, and associated patient safety issues. For nurses, ADC use can help improve medication safety, ensure pharmacists review orders prior to administration, and reduce or eliminate delays owing to medication availability, first-dose administration, missing doses, and time-consuming controlled substance counts. Pharmacists now spend less time dispensing drugs, and may have more time to collaborate with their nursing colleagues, check physicians’ orders against patients’ drug profiles, reconcile patient medication, participate in patient care rounds, and provide patient education.

The role of pharmacy technicians changed with the introduction of the ADC system. Nurses do not have to restock the ADC and manage medication expiry dates as this is done two to three times per week by the pharmacy technicians. The technicians have to go to the patient care unit to restock the ADC, a time-consuming activity, but one that supports better communication between the two departments. From a workload perspective, ADCs reduce pharmacists’ dispensing time, as inventory management is driven by the pre-established minimum and maximum levels and is handled exclusively by pharmacy technicians. Finally, the ADC system has improved compliance with many JCI standards around drug distribution, dispensing, and storage. It has built in methods to synthesize high-risk steps in the medication use process [10]. Profiled ADCs allow pharmacists to review and approve medication before it is available for selection and administration by the nurse, respiratory therapist, or physician. Computerized monitoring of drug administration to the patient by the nurse will provide accurate knowledge of patient medication history. This will help to optimize hospital drug distribution systems and enhance safe dispensing [11].

## Conclusions

This study explored nursing staff’s perceptions of and satisfaction with an automatic dispensing system in two specialized hospitals. The nurses were generally happy with the use of the ADCs over the 6 months following complete implementation and integration of the system. This may translate into better patient care and improved patient safety. These findings strongly support the government’s push to improve patient care. Future studies should focus on issues of patient safety, such as the system’s influence on medication errors, especially dispensing and administration errors.

## Additional file

Additional file 1:
**Automated Dispensing Cabinet Satisfaction Survey.** (PDF 99 kb)

## Abbreviations

ADC: Automated dispensing cabinets
HH: Heart Hospital
NCCCR: National Center for Cancer Care and Research (NCCCR)
HMC: Hamad Medical Corporation
ISMP: Institute for Safe Medication Practices
JCI: Joint Commission International
