# Supplementary material for: Nurses’ perceptions of and satisfaction with the use of automated dispensing cabinets at the Heart and Cancer Centers in Qatar: a cross-sectional study
Source: BMC Nurs. 2016 Jan 14;15:4. doi: 10.1186/s12912-015-0121-7 (PMC4712505; doi:10.1186/s12912-015-0121-7)
Supplement: Additional file 1: — Automated Dispensing Cabinet Satisfaction Survey. (PDF 99 kb) [file 12912_2015_121_MOESM1_ESM.pdf]

Automated Dispensing Cabinet Satisfaction Survey

This is a satisfaction survey concerns the use of automated dispensing cabinets (PYXIS) in relation to pharmacy services provided to nursing for patient care. Please circle the one that best represent your opinion. The information will be used for the purpose of improving the quality of medication delivery system.

What is your title:
◻ Staff Nurse ◻ Charge Nurse ◻ Head Nurse ◻ Other

Nursing Unit:----------------
Number of Beds:------------
Nurse Per Patient Ratio:----

A. Personal Details:

1. Nursing Degree:
◻ Diploma ◻ BSc Nursing ◻ MSc ◻ Other

2. Years of experience:
◻ 1 – 5 years ◻ 5 – 10 years ◻ 10 – 15 years ◻ More than 15 years

4. Have you worked with Pyxis before joining Heart Hospital?
◻ Yes ◻ No

B. System Use:

1. From logging in to actually withdrawing the medications you need, how easy is the process?
◻ Very easy ◻ Easy ◻ Somewhat easy ◻ Not easy

2. When have you started using Pyxis at the Heart Hospital?
◻ 1 – 3 months ◻ 3 – 6 months ◻ 6 – 9 month ◻ Never used the system

3. How would rate your satisfaction with PYXIS?
◻ Very satisfied ◻ Satisfied ◻ Somewhat satisfied ◻ Not satisfied

<u>C. Answer the following questions by choosing one of the following:</u>

- Strongly Agree - Agree - Disagree - Strongly Disagree

#	Question	Strongly Agree	Agree	Neutral	Disagree	Strongly Disagree
1	Medications are more readily available?	◻	◻	◻	◻	◻
2	The medication delivery system allows me to do my job more safely?	◻	◻	◻	◻	◻

Automated Dispensing Cabinet Satisfaction Survey

3	There are rarely discrepancies when doing narcotic counts.	◻	◻	◻	◻	◻
4	I have access to all of the medications I need.	◻	◻	◻	◻	◻
5	I am able to get all of my medications in one place.	◻	◻	◻	◻	◻
6	I rarely have to wait in line to get my patient medications.	◻	◻	◻	◻	◻
7	Refrigerated medications are easily accessible.	◻	◻	◻	◻	◻
8	It is easy to obtain medications during an emergency.	◻	◻	◻	◻	◻
9	The physical layout of the system is user friendly.	◻	◻	◻	◻	◻
10	All drawers' types assures safety access and removal of medications	◻	◻	◻	◻	◻
11	I can use the system confidently after minimal training.	◻	◻	◻	◻	◻
12	The training materials provided were informative & adequate.	◻	◻	◻	◻	◻
13	Did you view the PYXIS video prior to Go-Live?	◻	◻	◻	◻	◻
14	I was adequately trained by a PYXIS representative or a super-user nurse prior to Go-Live?	◻	◻	◻	◻	◻
15	The system would work better if more meds were in the PYXIS system?	◻	◻	◻	◻	◻
16	I am able to administer meds more efficiently (on time, right dose, etc.) with the PYXIS system.	◻	◻	◻	◻	◻
17	I am able to select the best					

	available strength of ordered medication					
18	I now spend less time waiting for medications that come from pharmacy than before PYXS was installed.	◻	◻	◻	◻	◻
19	The amount of time between when a written order is sent to Pharmacy and when it is available from the PYXIS system is acceptable.	◻	◻	◻	◻	◻
20	The number of phone calls to the pharmacy requesting missing medications is acceptable?	◻	◻	◻	◻	◻
21	Pharmacy personnel have been responsive in answering questions and/or resolving issues.	◻	◻	◻	◻	◻

D. Suggestions:

1. What do you like the most about PYXIS system?
1.
2.
3.

2. What do you like the least about PYXIS system?
1.
2.
3.

3. Do you have any suggestions for improving the system?
1.
2.
3.
